# Differentiation of Pulsatilla and Phosphorus, with Illustrative Observations*Read before The International Hahnemannian Association at Niagara, June, 1897.

**Published:** 1897-09

**Authors:** B. L. B. Baylies

**Affiliations:** Brooklyn, N. Y.


### DIFFERENTIATION OF PULSATILLA AND PHOSPHORUS,* WITH ILLUSTRATIVE OBSERVATIONS.

B. L. B. Baylies, M. D., Brooklyn, N. Y.

I do not know that the diagnosis of the two remedies, Puls. and Phos., will gratify the "grave and reverend" seniors of our Association, but may interest and encourage less erudite members and associates to studiously analyze and compare the related individuality of our materia medica. Such a study is not original or creative, but practical.

Phosphorus has an excitable temperament, and is easily angered, is often given to melancholy, languor, and apathy, yet sometimes seized with spasmodic laughing and weeping; has often frightful visions and hallucinations; at the approach of night, fear and apprehension of death; is disinclined to mental and physical work, slow of thought, and of difficult utterance; is physically weak, with numb and paralytic feeling of the hands and feet, heaviness of the lower limbs, slowness of motion and locomotion, sensitiveness over the spines of the dorsal vertebræ, the flexor muscles of the lower ex-

---

*Read before The International Hahnemannian Association at Niagara, June, 1897.

tremities inclined to contract, and painful on pressure; sensation of great weakness across the umbilicus, and in the lower abdomen.

Pulsatilla is of variable humor, often alternating smiles and tears; timid and fearful of ghosts, anxious about health and business, despondent and tearful when narrating her complaints, capricious, fretful or impatient, given to covetousness, is physically troubled with tremulous weakness, trembling with cold perspiration, stretching and yawning, drawing and jerking pains in the muscles, erratic pains in the joints, better by motion, is worse from lying on either side, especially on the left side; when worse from lying she is relieved by rising or sitting, but weariness and weakness often compel the recumbent posture; is predominantly better by change of position and motion, while Phosphorus is relieved by rest. Pulsatilla has fear of ghosts, but has not, like Phosphorus, frightful visions and hallucinations.

A young married woman having seen an insane person, was greatly terrified by the recollection, with fears that she herself would become insane. Becoming pregnant, she had, during gestation, quite profuse monthly hemorrhages of dark and clotted blood, which caused her much apprehension, with weeping and trembling. On each occasion Pulsatilla quickly arrested the flow, a cheerful and normal state of mind returned, and a healthy child was born at full term.

Phosphorus has weakness of head, heavy, dull, stupid, numb, and dizzy feeling, or bruised and beaten sensation of the brain, burning and fullness of the head. Both Pulsatilla and Phosphorus have stitching pains, Phosphorus in the temples, sides of the head, crown, and occiput; Pulsatilla in the forehead, temples, and occiput. The stitching pains of Pulsatilla may extend from the occiput through the ears. Both have pulsation and pressure in various parts of the head, Phosphorus pressure alternately in the temple and top of the head; both, pulsation in the occiput, that of Phosphorus, on rising after stooping; that of Pulsatilla is a severe hammering. Pulsatilla has rhythmical throbbing, beating of the pulse in head distinctly heard. Most distinguishing characteristic feelings of Phosphorus are the numb, stupid, bruised and beaten feeling of the brain, burning and fullness of the head. Both have vertigo and staggering while walking, as if intoxicated; Pulsatilla has the peculiar symptoms, confusion and hollow feeling in the head. The head felt like a lantern; also headache, as if one had eaten too much, causing disorder of the stomach; both feel better in the open air. Phosphorus is relieved of a pressive one-sided headache by walking in the open air. The hair of Phosphorus is painfully sensitive to the touch; there is sensitiveness at the vertex, as though some one were pulling the hair; the hair falls. Pulsatilla and Phosphorus are better when lying with head high, Pulsatilla so, predominantly.

I will not attempt to describe all their various affections of the eye, remarking, however, the twitching eyelids common to both. A peculiar sensation of Pulsatilla, which reminds us of Crocus-sativa, is as if mucus were hanging before the eye that obscured the vision, and that could be wiped away. Phosphorus has icteric hue of the conjunctiva, sees better when the pupils are dilated by shading the eyes with the hand, suggesting the ability of Phosphorus to cure cataract.

A lady of sixty-five years, a patient of the writer, was healed of cataract by Phosphorus, the diagnosis of her case having been confirmed as such by a well-known oculist.

Pulsatilla is troubled with redness and swelling of the external ear, pain in the ear, as if something were forcing outward, jerking in the ears; itching and stitching in the inner ear. Phosphorus also, pain in the external ear, better from pressure, feeling of pressure in both ears, burning and violent stitches through the ear and teeth at night. Pulsatilla has murmuring in the ear rhythmical with the pulse, difficult hearing, as if the ears were stopped. Phosphorus hyperæsthesia of all the senses, especially of hearing and smell, reverberation of all tones spoken in the same pitch; on waking from sleep, sound of words seems too loud. Pulsatilla re-echo in the ear, cracking in the ear on moving the head or body, tinnitus aurium, as in cinchonism.

Phosphorus has frequent sneezing, sneezes spasmodically, with violent pain in the head, distortion of the limbs, and constriction of the chest. Sneezing aggravates the unpleasant *stuffed* feeling of the chest. Nose is swollen and painful to the touch, nostrils internally inflamed or ulcerated, sensation of dryness with bleeding, sneezing in the morning, with tearing pains in the throat; watery coryza in the open air. Pulsatilla, sneezing in the evening in sleep, in the morning in bed, coryza with impaired smell and taste, epistaxis, blowing blood from the nose in the morning; Phosphorus, frequent alternation of fluent and stopped coryza, frequent sneezing, with pressive pain in both sides of the head; profuse epistaxis; later attacks attended by profuse sweat; bleeding of the nose several times a day, blowing of blood and yellow mucus from the nose, and mucus streaked with blood. This condition occurring in the second stage of acute catarrh. Phosphorus easily arrests, but does not prevent its recurrence—a disappointment to physician and patient. Satisfaction can only arise from abortive treatment in the first stage. Pulsatilla has green, offensive discharge from the nose, purulent discharge from the right nostril, stopped catarrh with ulcerated nostril, stoppage in the evening; in the morning, thick, yellow mucus blown from the nose. Phosphorus, stoppage alternately right or left, evening and morning, so that she could only breathe with the mouth open; with pain extending to the middle of the forehead. Pulsatilla, pressive sensation in the root of the nose, pains in the bones of the nose. Phosphorus is especially sensitive to offensive odors. Pulsatilla has smell as of old catarrh in the nose, fictitious smell of tobacco and coffee. The idiopathic hemorrhages of the nose, lungs, or stomach to which Pulsatilla corresponds, are often vicarious of suppressed menses. Those related to Phosphorus result from acute local hyperæmia, or from degeneration of the tissues; ulcers, necrosis of bones or blood-vessels, as in apoplexy and purpura hæmorrhagica. A man fifty-eight years of age, exsanguineous, sallow, and cachectic, feeble, and short-breathed from slight exertion, partially hemiplegic on the right side, had almost constant laryngeal cough, with each cough expectorating bloody, frothy mucus, and blowing similar matter from the nose; the lower limbs felt heavy and clumsy; auscultation showed dilatation and atrophy of the heart. Phosphorus arrested the cough and hemorrhages, and reduced the paralysis on several occasions, but over-exertion and anxiety increased the weakness of the heart, hastening death by pulmonary apoplexy. Both Phosphorus and Pulsatilla have paleness of the face; Phosphorus, the hippocratic face, sunken, with anxious look, and deep hollow eyes, surrounded by blue rings; or, the face may be red and congested or icteric. Puls. has sudden redness of the face, or redness and burning of the right cheek, more in the open air; swelling of the lower lip, cracked in the middle; Phos. —lips dry and parched, lower lip deeply cracked in the middle; both have twitching of the facial muscles: Puls., twitching of the lower lip, drawing, jerking pains in the lower jaw; Phos., trismus, closure of the jaws so that he could not separate the teeth; the gums bleed easily from the slightest touch, and ulcerate; drawing, jerking toothache, from cool air or taking anything warm into the mouth; carious teeth, teeth fall out, necrosis of the upper or lower jaw. Puls. has drawing, jerking, or sticking, digging pains in the teeth, changing from one jaw to the other, from taking anything very *warm* or *cold*, by warmth of bed, by chewing; at 6 to 11 P. M., better by draft of cool open air; sore gums, throbbing of the gum rhythmical with the pulse. The throat is covered internally with tenacious mucus in the morning; dryness of the throat and mouth extends to the tip of the tongue. Phos. has much coughing, expectorating gray, salty mucus in the morning; cough worse from singing, mucus hawked up in the morning is cool. Puls. has dryness of the throat and mouth, with sensation on swallowing as though the throat were constricted posteriorly and closed; dysphagia, as from paralysis of the pharynx. Phosphorus, throat sore, as if grown together, while swallowing or not swallowing; pain in the larynx on external pressure. Puls., sensation of clawing and scraping in the throat as after violent vomiting, not felt while swallowing. Sensation of swelling in the throat and rawness in the trachea on swallowing. Phos., sweet taste in the throat with accumulation of saliva, acidity in the throat, and scraping in the pharynx, rawness and soreness in the throat with dark redness posteriorly; throat sore, as from swelling of the uvula.

Puls., sensation of swelling, sometimes in the upper, sometimes in the lower throat; on swallowing, pain as though the uvula were swollen, sensation as if something acrid were on the palate, as if the palate were raw on swallowing. Phos., burning in the throat and nose, down the throat, and in the stomach; rawness of the throat, with violent coryza; pain in the throat when sneezing and yawning; pressure in the throat extending to the stomach. Puls. has burning in the pharynx after vomiting; stitches in the throat when not swallowing, none when swallowing; scraping and dryness in the throat, causing paroxysms of two or three coughs; sensation as of Sulphur vapor in the throat while coughing. Phos., scraping of the throat provokes cough in the open air; violent sticking irritation to cough; pressure in the throat, with swelling of the tonsils, the touch of which causes a hacking cough; great swelling of the tonsils; tonsils enlarged with much dysphagia; swelling of the left tonsil, impeding motion of the head. Puls., attacks of constriction, or retching pain in the pharynx, as if one had swallowed too large a morsel of bread; sensation as of a worm creeping up in the throat. Phos., fullness in the upper pharynx, as if the food stood high up, and must be vomited, without nausea. Puls., dysphagia, as from paralysis of the pharyngeal muscles. Phos., parotid gland tense and painful on stooping, painful to the touch; burning in the parotid gland, throbbing in the carotids. Puls., boring pain, drawing tension in the submaxillary glands.

Without further extending, at this reading, the parallel or contrasting symptoms of Phosphorus and Pulsatilla, I conclude with a few practical observations. The two remedies have in common some very prominent traits: both are disposed in general to lie upon the right side, which may be attributed possibly to their irritative influence upon the nerves of the stomach or heart; the sensation, burning soreness and fullness, nausea, eructations, and vomiting of Phos. are accompanied by craving thirst for copious cold drinks, ejected as soon as they become warm in the stomach; while the gastric burning, eructation, mucous-bilious vomit of Puls., are frequently unattended by thirst or local soreness. Both, if in bed, rise to the sitting posture when coughing; Puls. to relieve threatened suffocation attending the cough, and aid expectoration; Phos., to rerelieve soreness, tightness, and stitches in the middle of the chest, sometimes extending from the sternum to the interscapular space. It assists this relief from posture, by pressure of the chest with the hand.

A pregnant woman with albuminuria and general anasarca was troubled immediately on lying down with burning in the stomach, chest, and throat, and cough that compelled her to sit up in bed to prevent suffocation. Puls. removed these symptoms, gradually increased the quantity of urine from seven to eighty ounces daily, with proportionately diminished albumin, and restored her to health. Puls. has muscular, jerking, and twitching pains worse when lying on either side, palpitation worse when lying on the left side, headache worse when lying with the head low, stitching, drawing pains like labor pains, extending from the lower back upward and forward through the abdomen, from the back forward, terminating with stitches in the region of the stomach.

The case of peritonitis and appendicitis which I reported at the last meeting as cured by Pulsatilla, presented these peculiar pains between the back and abdomen, and other characteristics, with the conditions of aggravation or amelioration, as to position and periodicity, belonging to this remedy.

